# Significance of a Higher-Than-Expected Resonance at a 2.02 ppm Chemical Shift on 1H-Magnetic Resonance Spectroscopy (1H-MRS) in Neuroimaging

**DOI:** 10.7759/cureus.97810

**Published:** 2025-11-25

**Authors:** Shumyla Jabeen, Richa S Chauhan, Shiva Shankar M P, Karthik Kulanthaivelu, Chandrajit Prasad, Maya D Bhat, Prasad Hanagandi, Dwarakanath Srinivas

**Affiliations:** 1 Department of Radiodiagnosis and Imaging, Sher-i-Kashmir Institute of Medical Sciences, Srinagar, IND; 2 Department of Radiodiagnosis, All India Institute of Medical Sciences, Raipur, Raipur, IND; 3 Department of Radiodiagnosis, Tagore Medical College and Hospital, Chennai, IND; 4 Department of Neuroradiology and Interventional Radiology, Sri Ramachandra Institute of Higher Education and Research, Chennai, IND; 5 Department of Neuroimaging and Interventional Radiology, National Institute of Mental Health and Neurosciences (NIMHANS), Bangalore, IND; 6 Department of Neuroimaging and Interventional Neuroradiology, National Institute of Mental Health and Neurosciences (NIMHANS), Bangalore, IND; 7 Department of Radiology, King Abdulaziz Medical City, Ministry of National Guard Health Affairs, Riyadh, SAU; 8 Department of Neurosurgery, National Institute of Mental Health and Neurosciences (NIMHANS), Bangalore, IND

**Keywords:** colloid cyst, magnetic resonance spectroscopy, mucinous, neurenteric cyst, salla’s disease

## Abstract

Background: N-acetyl aspartate (NAA) is a commonly evaluated neuronal metabolite in ^1^H-Magnetic Resonance Spectroscopy (^1^H-MRS). The resonance at a specific chemical shift, although typically attributed to NAA, may reflect contributions from other N-acetyl-containing compounds.

Purpose: This study sought to identify the neuroimaging substrates with a greater-than-expected prominence of the singlet peak at 2.02 ppm in ^1^H-MRS across various conditions and to explore their biochemical basis. The operational definition was a conspicuously tall singlet at 2.02 ppm whose amplitude exceeded the expected NAA peak relative height of choline and creatine peaks for that anatomical region and acquisition TE (time to echo).

Materials and methods: A retrospective descriptive analysis was performed on the institutional imaging database. The search terms for identifying entities included "elevated NAA", "prominent NAA", and "elevated peak at 2.02 ppm". The clinical and imaging features were recorded for eight cases that fulfilled the search criteria.

Results: Eight entities fulfilled the search criteria in retrospective analysis. The conditions were Canavan's disease, Pelizaeus-Merzbacher disease, Pelizaeus-Merzbacher-like disease, Salla's disease, colloid cyst, neurenteric cyst, tumoral cyst, and mucinous adenocarcinoma metastasis. A higher-than-expected/elevated peak at 2.02 ppm chemical shift at the predicted location of NAA in ^1^H-MRS was noted in these cases, all of which had an N-acetylated molecule in the pathological milieu.

Conclusion: An accentuated 2.02 ppm peak on ¹H-MRS should not be interpreted as NAA-specific without contextual correlation. The chemical shift of 2.02 ppm is a metabolite signature of the methyl protons of the N-acetyl group and provides no further information about attached moieties. The signal may arise from a wide gamut of compounds with N-acetyl groups arising from diverse biochemical substrates in metabolic, cystic, and neoplastic lesions, as elucidated. Awareness of these mimics improves diagnostic accuracy and prevents misinterpretation of spectroscopy findings.

## Introduction

N-acetyl aspartate (NAA) is an exclusive and specific marker for the neuronal population in the adult human central nervous system (CNS). NAA is considered a critical link between neuronal mitochondrial energy production and oligodendrocytic lipid synthesis in the human brain [1]. Evidence is now accumulating for many other putative roles of NAA, including the synthesis of NAA-glutamate (the most concentrated neuropeptide), osmoregulation, axon-glial signaling, and brain nitrogen balance. Relative decreases in the amplitude of the peak are the subject of numerous CNS pathologies. In ^1^H-Magnetic Resonance Spectroscopy (^1^H-MRS), the 2.02 parts per million (ppm) resonance is designated as NAA until proven otherwise. Neuroscience research into NAA metabolism has largely been driven by its distinctive 2.02 ppm signal in ^1^H-MRS and its association with the rare, usually fatal Canavan's disease.

Earlier, MR spectroscopy literature had far exceeded the basic sciences literature on NAA. The incongruity in these bodies of NAA research has hampered the correct interpretation of the so-called "NAA peak" in many disease states. Given its ubiquitous presence, there exists a need for a radiopathological and radio-biochemical correlation for the apparently "elevated NAA" peaks in rare but diverse clinical contexts.

Herein, our research question is: Which neuroimaging entities exhibit a higher-than-expected NAA-like resonance at 2.02 ppm, and what are the likely biochemical correlates underlying this finding? We aim to identify pathological conditions that exhibit a prominent NAA-like peak on ¹H-MRS and to explore the biochemical correlates responsible for this resonance. Thereby, we intend to address an important interpretative gap in clinical MRS by expanding the framework for understanding elevated N-acetyl signals.

## Materials and methods

Type of study

This is a descriptive observational study from a tertiary care referral center in South India. This retrospective study was conducted in accordance with standard institutional protocol.

Subjects

The institutional Picture Archiving and Communication System (PACS) database was queried for brain MRI examinations performed between January 2012 and December 2021. All studies containing spectroscopy were searched in the PACS database at the institution. The search terms included "elevated", "prominent", "marked", "NAA", and "2.02 ppm". Clinical details were obtained from the medical records section. After review of spectra and voxel placement, eight cases met the final inclusion criteria. Inclusion criteria were (1) presence of a conspicuously elevated singlet at 2.02 ppm on ¹H-MRS; (2) voxel of interest (VOI) convincingly placed within the lesion; (3) adequate spectral quality; and (4) availability of genetic/histopathological confirmation. Spectra with poor signal-to-noise ratio, contamination, or lack of final histopathological/genetic confirmation were excluded. Leukodystrophy cases were restricted to genetically confirmed diagnoses to improve internal validity.

Imaging technique and analysis

Imaging was carried out on one of two scanners: a 1.5 Tesla Siemens (Aera, Siemens Medical Systems, Erlangen, Germany) or a 3 Tesla (Ingenia, Philips Medical Systems, Best, The Netherlands). Besides conventional structural imaging sequences, these cases underwent 2D multivoxel ^1^H-MRS using Point-Resolved Spectroscopy (PRESS) with automatic water suppression (water suppression by excitation) at an intermediate TE (time to echo) of 135-144 milliseconds. This single-slice spectroscopy sequence employs a spin-echo scheme for volume selection to eliminate chemical shift displacement. The acquired voxel dimensions were 10x10x15 mm. This intermediate TE was chosen such that J-coupled signals are in phase, and the lactate doublet, if present, is in the opposed phase of the spectrum.

B0 homogeneity was achieved by pencil beam auto-shimming. The repetition time (TR) was 1500 ms for Aera and 2000 ms for Ingenia, with the number of averages = 4. The TRs were chosen to allow sufficient T1 relaxation and reduce the saturation effects. A half-echo acquisition mode was adopted to allow for phase correction. The spectral resolution achieved was 1.95 Hz/p (Table 1). Care was taken to ensure that the voxel of interest was indeed representative of the lesion with no contamination/bleeding for adjacent normal/uninvolved parenchyma. Areas of hemorrhage were excluded for voxel positioning. Post-processing involved spectral correction with no correction for shifted metabolite displacement. Quantification was not performed. Raw spectroscopy data were not archived in the PACS during the study period; only processed screenshots were available, precluding retrospective reprocessing or model-based quantification. Snapshots of the fitted/processed spectra were analyzed, and a peak was identified at 2.02 ppm; its relation to other metabolite peaks was reviewed. All the cases were genetically or histopathologically confirmed.

**Table 1:** Sequence parameters for spectroscopy PRESS: point-resolved spectroscopy; TE: time to echo; TR: repetition time; WET: water suppression enhanced through T1 effects

Parameter	Siemens Aera 1.5T	Philips Ingenia 3T
Sequence	PRESS	PRESS
TE	135 ms	144 ms
TR	1500 ms	2000 ms
Averages	4	4
Water suppression	WET	WET
Shimming	Pencil-beam	Automatic B0
Voxel size	10×10×15 mm	10×10×15 mm

## Results

The following are the entities in which a higher-than-expected peak was identified at 2.02 ppm.

Case I: Canavan's disease

**Figure 1:**
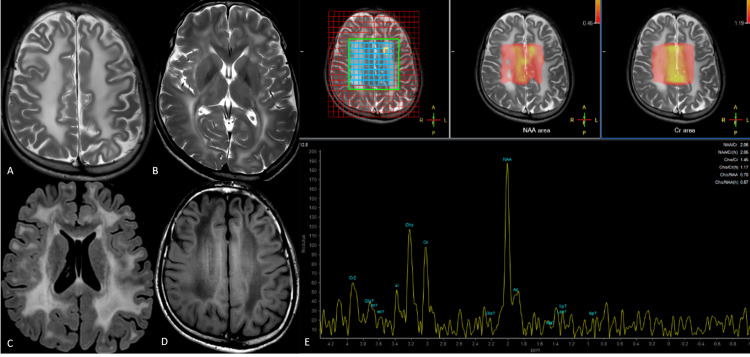
Canavan's disease Axial T2-weighted images above (A) and at the level of the basal ganglia (B) show confluent hyperintensity involving the subcortical white matter extending deep into the periventricular white matter. The internal capsules are involved with the sparing of the corpus callosum. An axial FLAIR image at the level of the basal ganglia (C) shows hyperintense white matter with a signal inversion in the bilateral centrum semiovale (D). The MR spectra from the involved white matter (E) show a markedly elevated NAA peak suggestive of Canavan's disease. FLAIR: fluid-attenuated inversion recovery; NAA: N-acetyl aspartate

An 18-year-old boy presented with seizures, psychomotor delay, and progressive walking difficulty. MRI revealed extensive signal changes, including T1 hypointensity and T2/FLAIR (fluid-attenuated inversion recovery) hyperintensity, involving the subcortical white matter and extending into the periventricular region. The corpus callosum, bilateral caudate nuclei, and putamen were relatively spared (Figure 1).

Multivoxel MRS at intermediate TE from the involved white matter in the centrum semiovale revealed a tall, conspicuous peak at 2 ppm with a relatively reduced height of choline and creatine peaks. There was no evidence of a lactate peak. The involvement of the subarcuate fibers and the prominent resonance at 2.02 ppm, suggestive of the NAA peak, strongly favored the final diagnosis of Canavan's disease in this case. Genetic analysis for a mutation in the *ASPA* gene confirmed the diagnosis.

Case II: Pelizaeus-Merzbacher disease (PMD)

**Figure 2:**
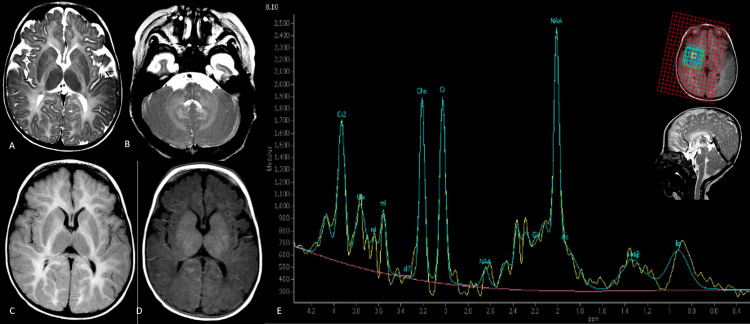
Pelizaeus-Merzbacher disease (PMD) Axial T2-weighted images show diffuse hyperintensity involving bilateral cerebral (A) and cerebellar white matter, with the involvement of bilateral dentate nuclei (B). Hyperintense white matter is noted on the axial FLAIR image (C). A closer look at the axial T2-weighted image (A) shows hypointensity in the posterior limb of the internal capsules, which is relatively hyperintense on T1 IR (D). Multivoxel MRS at intermediate TE with the voxel of interest placed over the basal ganglia and adjoining involved white matter (F) shows a conspicuous singlet peak at 2.02 ppm with reduced choline resonance. The inset in (F) above shows the voxel of interest, with the inset below showing the thinned-out corpus callosum on a sagittal T2-weighted image. Genetically proven PMD. FLAIR: fluid-attenuated inversion recovery; TE: time to echo; MRS: magnetic resonance spectroscopy

An eight-month-old boy presented with global developmental delay and nystagmus. An MRI of the brain showed a homogenous T2 hyperintense signal in bilateral cerebral and cerebellar white matter and both dentate nuclei (Figure 2).

An area of T2 hypointensity was seen in the posterior limb of the internal capsule, which was hyperintense relative to the rest of the white matter on T1 inversion recovery, representing a myelinated area. The corpus callosum was markedly thin and hyperintense. ^1^H-MRS revealed a high NAA-like peak at 2.02 ppm with reduced choline. The creatine peak was conspicuous. The lactate peak was absent. Homogenously hyperintense cerebral white matter with a prominent NAA peak was suggestive of a diagnosis of PMD. Genetic analysis confirmed the presence of duplication involving the segment of the X chromosome containing the *PLP1* gene.

Case III: Pelizaeus-Merzbacher-like disease (PMLD)

**Figure 3:**
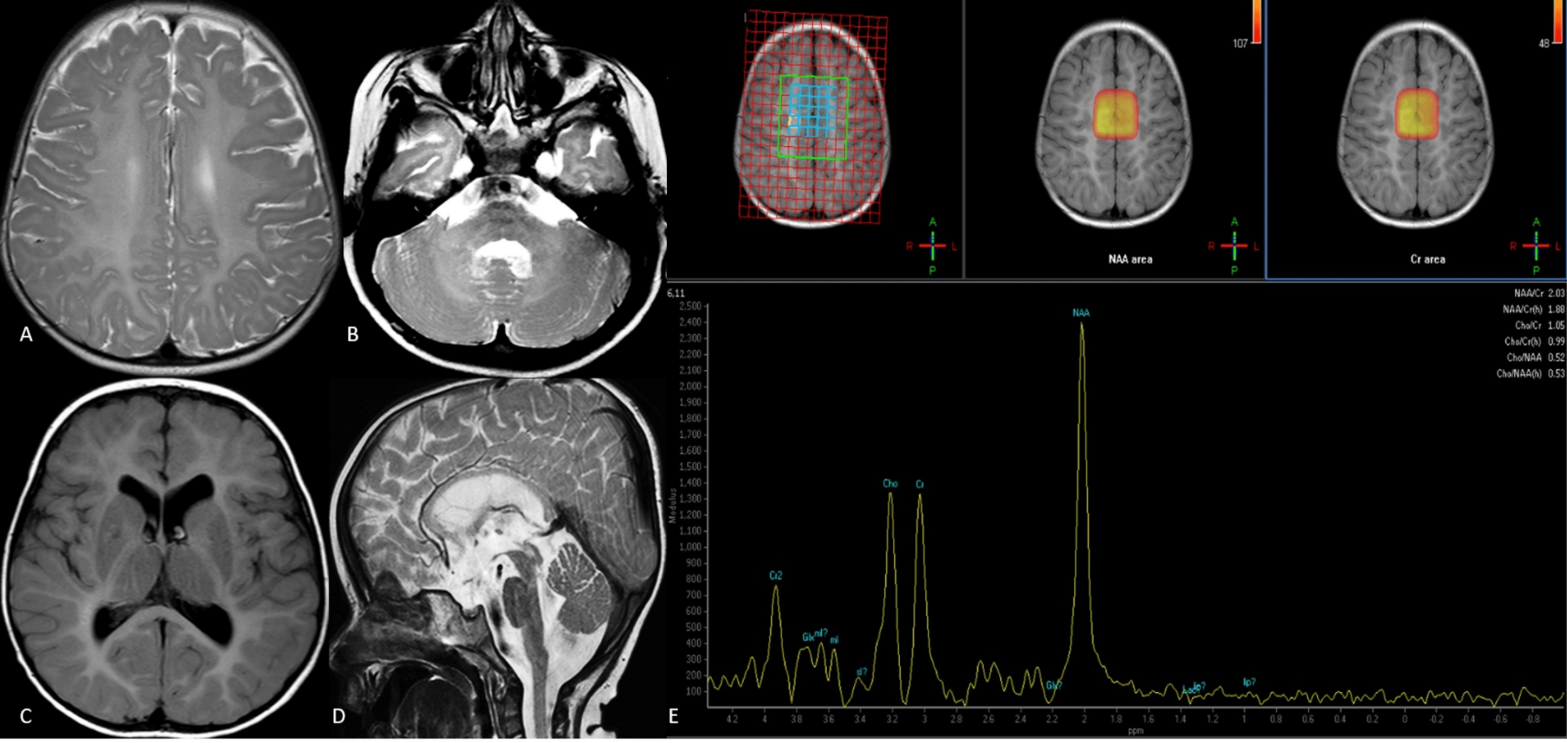
Pelizaeus-Merzbacher-like disease (PMLD) Axial T2-weighted images of a case of PMLD above the level of the basal ganglia (A) show confluent hyperintensity involving the entire white matter. The cerebellar white matter is also T2 hyperintense (B), with hyperintensity also noted in the pons (B). On axial FLAIR images (C), the white matter is hyperintense. A thin corpus callosum with a hyperintense signal is evident on sagittal T2-weighted images (D). Note the prominent NAA-like peak at 2.02 ppm with reduced choline on the MR spectra from the basal ganglia and adjoining white matter (E). FLAIR: fluid-attenuated inversion recovery; MR: magnetic resonance

An MRI of the brain of a two-and-a-half-year-old boy with motor developmental delays, ataxia, and spasticity demonstrated diffuse signal changes. T2 hyperintensity of bilateral cerebral and cerebellar white matter, with T2 hyperintense areas in the pons and corpus callosum, was seen (Figure 3).

^1^H-MRS from the involved white matter showed an elevated peak at 2.02 ppm with reduced choline. Hypomyelination with signal changes in the brainstem and a resonant peak at 2.02 ppm suggested a diagnosis of PMLD.

Case IV: Salla's disease

**Figure 4:**
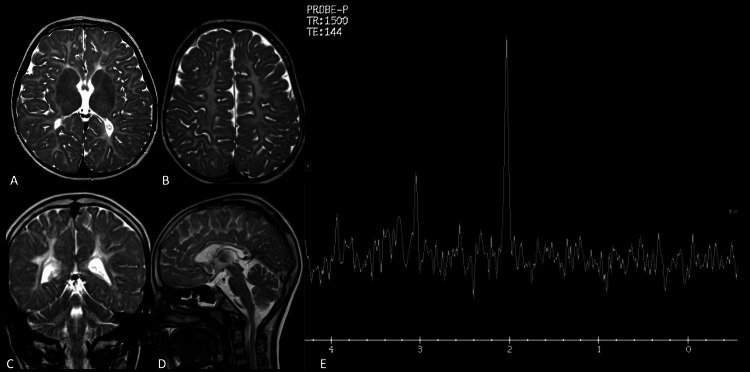
Salla's disease Confluent white matter T2 hyperintensity is visualized above (A) and at the level of the basal ganglia (B). The subcortical white matter is predominantly involved. Note the relatively hypointense basal ganglia and thalami on the axial T2-weighted image (B). The coronal T2-weighted image (C) shows symmetrical hyperintensity in the supratentorial and cerebellar white matter. The sagittal T2-weighted image (D) shows a thinned-out corpus callosum. Proton MRS at an intermediate TE (E) is remarkable for a prominent peak at 2.02 ppm, which is due to N-acetylneuraminic acid in this case of Salla's disease and closely resembles the NAA peak. TE: time to echo; NAA: N-acetyl aspartate

A nine-year-old boy presented with nystagmus, cognitive impairment, and walking difficulty. An MRI of the brain showed mild cerebral and cerebellar atrophy with diffuse hypomyelination. The corpus callosum was atrophied, and bilateral dentate nuclei showed T2/FLAIR hyperintensity (Figure 4).

The basal ganglia and thalami appeared relatively T2 hypointense. Hypomyelination with T2 hypointensity in the basal ganglia and thalami raised suspicion of a storage disorder. An elevated peak at 2.02 ppm with a prominent creatine peak and a less conspicuous choline peak was detected on the single-voxel ^1^H-MRS from the basal ganglia, which further added to the possibility of a lysosomal storage disorder. Increased urinary levels of free sialic acid suggested the diagnosis. The genetic diagnosis was confirmed by identification of the *SLC7A5* variant c.406A>G p. mutation.

Case V: colloid cyst

**Figure 5:**
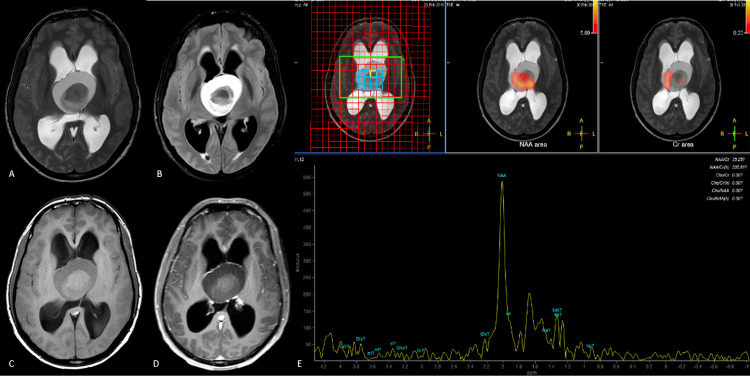
Colloid cyst The axial T2-weighted image (A) shows a well-defined, rounded, thin-walled cystic lesion in the third ventricular region leading to obstructive hydrocephalus. Note the hyperintense periphery with central T2 hypointensity (dot sign). Similar signal characteristics are seen on the axial FLAIR image (B). On the axial T1-weighted image (C), the cyst shows a central area of T1 shortening with the hypointense periphery. No internal enhancement is seen on the post-contrast T1-weighted image (D). The MR spectra at intermediate TE (E) from a voxel placed within the cyst show a large NAA-like peak at a chemical shift of around 2 ppm. Note the absence of other metabolites like choline and creatine. A doublet lactate peak is also seen at 1.3 ppm. An NAA-like peak in a cyst with the above imaging characteristics is strongly suggestive of a colloid cyst confirmed on histopathology. FLAIR: fluid-attenuated inversion recovery; NAA: N-acetyl aspartate; MR: magnetic resonance; TE: time to echo

A 30-year-old male presented with headache, vomiting, and blurring of vision. An MRI of the brain showed a large, well-defined, rounded third ventricular lesion extending up to the septum pellucidum, causing obstructive hydrocephalus. The lesion demonstrated a central T1 hyperintense and a profoundly T2 hypointense focus. The remaining portion of the lesion was hypointense on T1-weighted images and hyperintense on T2/FLAIR-weighted images (Figure 5).

There was no evidence of diffusion restriction or blooming. The lesion was non-enhancing on post-contrast enhancement. On multivoxel ^1^H-MRS, a large metabolite peak resembling NAA was detected at a chemical shift of 2.02 ppm with no discernible choline and creatine peaks and a doublet peak S/O lactate at 1.3 ppm. The central T2 hypointensity (dot sign) with an NAA-like peak suggested a diagnosis of colloid cyst, confirmed on histopathology.

Case VI: neurenteric cyst

**Figure 6:**
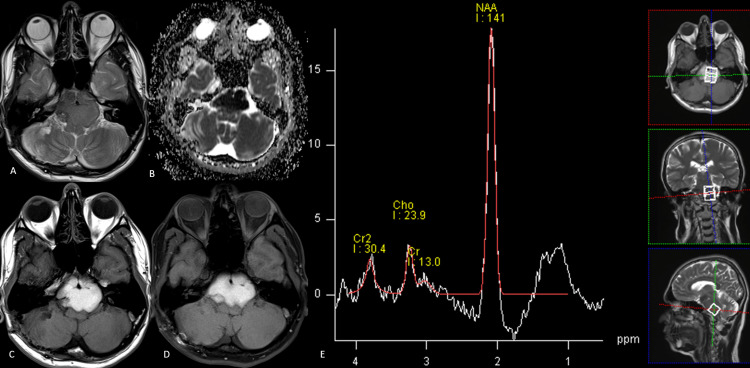
Neurenteric cyst A lobulated hypointense lesion is seen around the basilar artery, ventral to the medulla, extending into bilateral cerebellomedullary cisterns on the axial T2-weighted image (A). There is no narrowing or displacement of the vessel. ADC map (B) shows diffusion restriction. It is uniformly hyperintense on the axial T1-weighted image (C) with no loss of signal on the axial T1 fat-saturated sequence (D). The large NAA-like peak on MRS (E) supported a diagnosis of a neurenteric cyst, ruling out a white epidermoid. A lactate peak is also present. NAA: N-acetyl aspartate; ADC: apparent diffusion coefficient

A 24-year-old male presented with headache and right facial nerve palsy. An MRI of the brain revealed a large, midline lobulated T1 hyper- and T2 hypointense lesion anterior to the brainstem insinuating into the basal cisterns, reaching superiorly up to the foramen of Monroe, and causing obstructive hydrocephalus. It remained hyperintense on the T1 fat-saturated sequence (Figure 6).

Diffusion restriction was noted with no evidence of blooming. ^1^H-MRS showed an elevated NAA-like peak at 2 ppm with minimal choline and evidence of a lactate peak. Although conventional MRI raised suspicion of a neurenteric cyst and a white epidermoid, the view of diffusion restriction and a high NAA-like peak essentially ruled out the diagnosis of the latter, which is characterized by an amino acid peak and the absence of NAA.

Case VII: tumoral cyst

**Figure 7:**
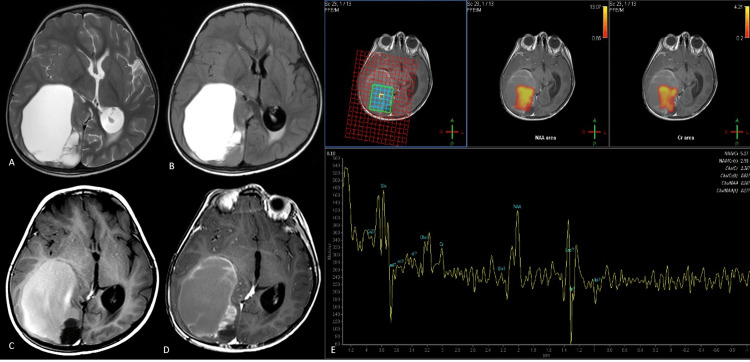
Tumoral cyst Axial T2-weighted image (A) shows a large hyperintense predominantly cystic lesion with a small solid component in the right temporooccipital region, causing uncal herniation. A small area of inversion is seen posteriorly on the axial FLAIR image (B), with the rest of the lesion retaining hyperintense signal. The cyst contents are predominantly T1 hyperintense (C). The post-contrast T1-weighted image (D) shows peripheral enhancement. The spectra (E) from a voxel located within the cystic lesion show a peak at a chemical shift of 2 ppm, with absent choline and creatine. A lactate peak is evident. Histopathology revealed a diagnosis of anaplastic ependymoma. FLAIR: fluid-attenuated inversion recovery

A six-year-old female presented with headache, seizures, and features of raised intracranial tension. MRI exhibited a mixed-signal intensity heterogeneously enhancing solid-cystic mass lesion in the right temporooccipital region, causing ipsilateral uncal herniation. The cystic component was T1- and T2-hyperintense, with no inversion on FLAIR. Mild perilesional edema was observed. ^1^H-MRS from the cystic component showed a peak at a chemical shift of 2 ppm along with a doublet lactate peak at 1.3 ppm (Figure 7).

There was no discernible choline or creatine peak. Metabolites suggestive of a parasitic cyst, such as succinate and pyruvate or abscess (amino acids), were absent, making the possibility of the neoplastic lesion more plausible. Histopathology revealed a diagnosis of anaplastic ependymoma.

Case VIII: metastasis

An MRI of a 40-year-old man with clinical features dominated by cerebellar signs revealed a large T1 hypointense/T2 hyperintense, peripheral-enhancing lesion in the left hemicerebellum with mild perilesional edema. A crescentic rim of peripheral T2 hyperintensity was seen in the lesion, consistent with the "pool sign." Single voxel spectroscopy within the lesion at an intermediate TE of 144 ms (voxel dimensions of 1x1x1 cm) revealed a distinct resonance at 2.02 ppm corresponding to the N-acetyl group. Histopathology of the excised specimen revealed a metastatic lesion due to mucin-producing adenocarcinoma. A systemic evaluation revealed a large mass involving the pancreas with multiple lesions in the liver.

The results are summarized in Table 2.

**Table 2:** Summary of findings in the entities NAAG: N-acetylaspartylglutamate dipeptide; CC: corpus callosum; WM: white matter

Entity	Conventional Imaging Features	Semi-Quantitative MRS Findings	Interpretation of 2.02-ppm Peak	Likely Biochemical Substrate	Confirmation
Canavan disease	Diffuse subcortical T2 hyperintensity; relative sparing of caudate/putamen	Markedly elevated 2.02 ppm peak, Cho↓, Cr↓, no lactate	Elevated N-acetyl resonance due to ASPA deficiency	NAA accumulation	*ASPA* mutation
Pelizaeus–Merzbacher disease	Diffuse hypomyelination; thin corpus callosum	Prominent 2.02 ppm peak, Cho↓, Cr preserved	N-acetyl signal from abnormal myelin turnover	NAA + NAAG contribution	*PLP1* duplication
PMLD	T2 hyperintensity of WM, cerebellum, pons; thin CC	Tall 2.02 ppm peak with low Cho	Prominent N-acetyl resonance despite hypomyelination	NAAG/NAA mixture	*GJC2* mutation
Salla disease	Hypomyelination, T2 hypointense basal ganglia	Prominent 2.02 ppm peak, Cr↑, Cho↓	Mimics NAA peak; reflects free sialic acid	N-acetylneuraminic acid (NANA)	*SLC7A5* mutation
Colloid cyst	T1 hyperintense/T2 hypointense center; "dot sign"	Solitary 2.02 ppm peak, absent Cho/Cr, lactate present	N-acetylated mucinous glycoproteins	N-acetylglucosamine/galactosamine	Histology
Neurenteric cyst	T1 hyperintense, T2 hypointense lesion ventral to brainstem	High 2.02 ppm peak, minimal Cho, lactate present	Secreted glycoproteins producing N-acetyl signals	Foregut-derived mucins	Histology
Tumoral cyst (ependymoma)	Cystic-solid lesion; T1/T2 hyperintense cyst	Sharp 2.02 ppm peak, absent Cho/Cr, lactate present	N-acetylated macromolecules in cyst fluid	Sialic/hexuronic acid-containing compounds	Histology
Mucinous adenocarcinoma metastasis	T1 hypointense/T2 hyperintense mass with "pool sign"	Distinct 2.02 ppm resonance, Cho variable	Mucin-associated N-acetyl protons mimicking NAA	Mucin N-acetyl residues	Histology + systemic workup

## Discussion

NAA in the adult brain is localized exclusively to the neurons and their processes. First described by Tallan et al. in 1956 following chromatographic analysis of a protein-free extract of the feline brain [2], it is now documented as a ubiquitous amino acid derivative in the human brain, second only to glutamate. Brain concentrations of NAA are high, reaching up to 10 mM [3]. Determining up to ~7% of the neuron's osmolarity, it exhibits a steep transcellular gradient [4].

Neuronal aspartate and acetyl coenzyme A are the substrates for the synthesis of this CNS-specific metabolite [1]. L-aspartate N-acetyltransferase is the key enzyme in NAA biosynthesis [5]. The localization of the subcellular fraction for this enzyme is in the neuronal mitochondria or microsomes. Neuron-derived NAA is transported to the oligodendrocyte cytoplasm, where it undergoes cleavage by *ASPA*, relieving the acetate moiety for fatty acid/steroid synthesis and eventually myelin synthesis. NAA anabolism and catabolism are thus compartmentalized to neurons and oligodendrocytes, respectively [4]. Postnatal myelination proceeds through a synchronized increase in the synthesis of NAA in neurons and in the production of the NAA-cleaving, lipogenic enzyme, aspartoacylase (ASPA), in oligodendrocytes. Putative functions ascribed to NAA include (i) molecular pump/osmolyte for water homeostasis in the neuron-glia interface; (ii) source of acetyl precursors for myelin synthesis; (iii) parent molecule for N-acetylaspartylglutamate dipeptide (NAAG), the most abundant peptide neurotransmitter of the mammalian CNS; (iv) coupling role with neuronal mitochondrial metabolic functions; (v) the second line of defense to brain nitrogen balance [6]; and (vi) protein stabilization by its osmolyte function [7].

Although usually attributed to NAA, the ^1^H-MRS peak at 2.02 ppm also has contributions from other acetylated species, such as NAAG, N-acetylneuraminic acid, and background coupled glutamate-glutamine resonances. By far, NAAG is the dipeptide with the highest concentration in the brain, constituting as much as 25% of all N-acetylated compounds in the brain [8,9]. The postulated functions of NAAG include neurotransmission, modulation of other neurotransmitters [10], and cell signaling at the neuron-glia interface.

Canavan's disease is a neurodegenerative spongiform leukodystrophy resulting from a mutation of the aspartoacylase-encoding gene [11]. Markedly elevated NAA due to the carboxypeptidase deficiency is a specific tell-tale feature. Failure of the osmolyte/water molecular pump function of NAA and defective oligodendrocyte myelin synthesis due to NAA reduction have been hypothesized to result in the spongiform leukodystrophy. Neuroimaging is characterized by a subcortical-predominant, expansile T2 hyperintense, T1 hypointense signal. Relative sparing of the caudate and putamen, with the involvement of the globus and thalamus, is frequently described. Wittsack et al. demonstrated that the NAA:Ch ratio increase in Canavan's disease is not limited to the reduction of Ch-containing compounds but may be in part due to an absolute increase in the NAA secondary to the enzymatic deficiency [12].

PMD is an X-linked-recessive leukodystrophy arising from mutations in the gene encoding proteolipid protein (PLP) on the long arm of the X chromosome (Xq22). Hypomyelination and later axonal degeneration ensue. Although classically due to duplication of the region in chromosome Xq22 encoding the *PLP* gene, other infrequent gene-dosage-related causes include deletions, point mutations, and triplication [13]. It is the PMD subtype with PLP1 duplications, characterized by a quantitative increase in NAA on ^1^H-MRS. The raised NAA was postulated to arise from either secondary axonal involvement in the background of dysmyelination or oligodendrocyte progenitor proliferation or the death of mature oligodendrocytes by Takanashi et al. [14]. Of note, raised CSF levels of NAAG have been reported in PMD.

PMLD1 is another hypomyelinating leukodystrophy with a clinical neurological phenotype comparable to PMD in terms of psychomotor developmental delay, nystagmus, spasticity, and hearing impairment. It is an autosomal recessive disorder arising due to mutations in the *GJC2* gene that maps to chromosome 1 [15]. ^1^H-MRS of the brains of patients with PMLD1 is reported to be normal [16]. At least one case report of raised NAAG in CSF of a girl with PMLD1 exists, similar to our index case.

Salla's disease, a hypomyelinating leukodystrophy, is a recessively inherited lysosomal storage disorder notable for the lysosomal accumulation of free sialic acid (N-acetylneuraminic acid, NANA). The basal ganglia and thalami appear T2 hypointense relative to the white matter, as described earlier in our index case. Quantitative ^1^H-MRS in Salla's disease has revealed approximately a 34% increase in the white matter N-acetyl moiety resonance at 2.02 ppm in a study by Varho et al. [17]. Also, they observed no differences in the chemical shifts of NAA and NANA. This increased N-acetyl resonance at 2.02 ppm may then be attributed to the free increased NANA content, which in fact may overbalance a possible drop in the leukodystrophy-associated NAA concentration. The same study reported higher creatine and lower choline levels compared to age-matched controls.

Intracranial cysts of the neurenteric and colloid types, despite the acellular nature of their internal contents, have been reported to demonstrate resonance at 2.02 ppm reminiscent of that due to methyl bond protons of the N-acetyl moiety of NAA of neuronal ensembles. Periakaruppan et al., in their study of colloid-like cystic lesions outside the third ventricle, opined that the signal at 2.02 ppm likely denotes the glycoproteins produced by the lining of columnar epithelium in these colloid-like cystic lesions [18]. On conventional imaging, a central T2-hypointense area, referred to as the dot sign, is known to occur in colloid cysts, which was evident in our index case. In a similar histological context, Andre et al. highlighted the presence of similar mimics at 2.02 ppm masquerading as NAA in cases of frontal sinus mucoceles. They opined that the signal at 2.0 ppm in mucoceles free of neuronal tissues emanates from the N-acetyl (CH₃) protons from mucous glycoproteins (mucins). Mucin-associated sugars, viz., N-acetylglucosamine, N-acetylgalactosamine, and N-acetylneuraminic acid, could then be the candidate species of the 2.02 ppm chemical shift peak [19].

Foregut duplication cysts, anomalous lesions of the bronchopulmonary anlage, are lined with similar pseudostratified columnar epithelium with a characteristic secretion of proteinaceous fluid. Given the similar nature of the exocrine activity of the epithelia of these cystic lesions, it is not unreasonable to find similar resonances at the 2.02 ppm chemical shift on ^1^H-MRS. It has been reported in a case of mediastinal foregut duplication cyst [20]. Notably, ex vivo spectra of N‐acetylgalactosamine and N‐acetylglucosamine showed a similar peak at 2.02 ppm, reiterating the likelihood of these moieties as the source of the signal. Neurenteric cysts arise due to faulty partitioning of the notochordal plate and the primordial endoderm around the third week. These cystic mass lesions are outlined by epithelia similar to those in the gastrointestinal and respiratory tissue. Remarkably, ^1^H-MRS in these lesions shows a peak at 2.02 ppm, again reflective of possible contribution from NAA-like compounds [21].

Given the lack of neuronal elements, metastases are intuitively not expected to demonstrate an NAA peak on ^1^H-MRS. NAA/Cho in metastases has been demonstrated to be significantly lower compared to white matter (0.00 and 1.17 vs. 2.68 ± 0.56; P < 0.0001 in both) [22]. Extending the above-discussed notion, it seems reasonable that metastases from "mucinous" adenocarcinomas may indeed demonstrate an NAA-like peak. Proof for this hypothesis is available from work on intracranial metastatic mucinous adenocarcinomas by Liu et al., who demonstrated a large metabolite resonance at 2.0 ppm simulating the NAA peak of normal brain tissue [23]. Metastatic mucinous adenocarcinomas are also characterized by the "pool sign" on conventional T2-weighted images, which can provide a clue to the diagnosis, a finding that we noted in our index case [24].

LC model-driven processing with position fitting for NAA and Cr peaks in pilocytic astrocytoma has revealed that the chemical shift between the NAA and Cr was significantly smaller compared to controls. Tamrazi et al. opined that this chemical shift position is not concordant with NAA and may likely arise from other N‐acetylated sugars [25].

Tumoral cysts with no discernible neuronal fractions have been detected with a 2.03 ppm resonance on ^1^H-MRS. Ex vivo analyses of tumoral cyst fluid in the brain brought novel observations. The 2.03 ppm ¹H-MRS signal in tumoral cysts has been artifactually ascribed to NAA as a result of outer volume contamination. Quantitatively, only 34% of the equivalent of the total signal attributable to the 2.03 ppm chemical shift of the NAA could be retrieved with perchloric acid. This points to the contribution and existence of sialic acid and hexuronic acid bound to macromolecules. These acidic moieties are insoluble in perchloric acid and are therefore not extractable by conventional methods [26]. Although our case of the tumor-associated cyst was related to that of an anaplastic ependymoma, we presume that the prominent resonance at 2.02 ppm chemical shift would have probably arisen from a similar pathophysiology.

Limitations

We acknowledge the limitations of the study. The study was retrospective in nature. The raw data of the spectra were not available in PACS for reprocessing. Quantitation and ratio estimation with reference to the other metabolites could thus not be performed. Snapshots of representative spectra after post-processing were retrieved alongside the report database for the analysis. Nevertheless, care was taken during the review of the snapshot images to ensure that the intended voxel was representative of pathology, with no voxel contamination from the adjacent/uninvolved brain. The sample size of our study was small. We acknowledge that the PACS search terms ("prominent NAA", "elevated 2.02 ppm") are inherently enriched for positive cases and may have excluded borderline or unrecognized instances. Normal control spectra could not be matched because voxel locations varied widely across cases.

## Conclusions

In conclusion, this study broadens the interpretive framework of the 2.02 ppm resonance on ¹H-MRS by demonstrating that a prominent peak at this location can arise from multiple N-acetylated species beyond N-acetylaspartate. For neuroradiologists, these findings emphasize the need to interpret the 2.02 ppm peak within an anatomical and pathological context. In cystic lesions, the presence of an N-acetyl resonance should prompt consideration of mucinous or glycoprotein-rich etiologies. In leukodystrophies, the relative contributions of NAA, NAAG, and N-acetylneuraminic acid may provide biochemical clues to the underlying metabolic disruption.

Prospective studies incorporating raw spectral data, quantitative modelling, and metabolic correlation are needed to better differentiate individual N-acetyl components. By reframing the 2.02 ppm peak as a chemical signature of the N-acetyl group rather than an exclusive marker of NAA, this work offers a more nuanced approach to spectroscopic interpretation and encourages metabolically informed radiological assessment.
